# 2487. Which Extended-Spectrum β-Lactamase-Producing Enterobacterales Colonize the Gastrointestinal Tract of High-Risk Patients?

**DOI:** 10.1093/ofid/ofad500.2105

**Published:** 2023-11-27

**Authors:** Haley Stambaugh, Shawna Lewis, Emily B Jacobs, Dariusz A Hareza, Yehudit Bergman, Sara E Cosgrove, Pranita Tamma, Patricia J Simner

**Affiliations:** Johns Hopkins School of Medicine, Cockeysville, Maryland; Johns Hopkins University, BALTIMORE, Maryland; Johns Hopkins School of Medicine, Cockeysville, Maryland; Johns Hopkins University, BALTIMORE, Maryland; Johns Hopkins, Baltimore, Maryland; Johns Hopkins School of Medicine, Cockeysville, Maryland; Johns Hopkins School of Medicine, Cockeysville, Maryland; Johns Hopkins School of Medicine, Cockeysville, Maryland

## Abstract

**Background:**

The epidemic of extended-spectrum β-lactamase producing *Enterobacterales* (ESBL-E) continues to expand. ESBL-Es commonly colonize the intestinal tract and may propagate the spread of ESBL genes. However, the frequency at which ESBL production occurs in all *Enterobacterales* (including inducible AmpC-producers) is unknown. This study aims to define the epidemiology of third-generation cephalosporin resistant *Enterobacterales* (3GC-RE) colonization to understand their burden and contribution to ESBL spread in high-risk populations (e.g., ICU, oncology, transplant).

**Methods:**

Surveillance cultures for 3GC-RE were performed by collecting perirectal swabs among high-risk populations at The Johns Hopkins Hospital. Isolates were identified by MALDI-TOF MS after recovery on 3GC selective chromogenic media. Antimicrobial susceptibility testing was performed using lyophilized broth microdilution panels following Clinical and Laboratory Standards Institute (CLSI) guidelines. 3GC-RE were characterized using the CLSI ESBL disk test among recommended *Enterobacterales*. For inducible AmpC-producing *Enterobacterales*, cefepime +/- clavulanic acid disk test was performed. Carbapenem-resistant *Enterobacterales* (CRE) were tested for carbapenemase-production using the modified carbapenem inactivation method (mCIM) and by the CARBA 5 lateral flow assay, if mCIM positive.

**Results:**

Of 966 surveillance cultures, 99 (10%) were positive for 3GC-RE and 14 (1%) were positive for CRE. Almost all 112 (99%) rectal swabs had a single organism isolated while multiple organisms were isolated from 1 (1%). 114 bacterial isolates were recovered (Table 1). Of the 3GC-RE, 66 (58%) were ESBL-producers with *Escherichia coli*, *Klebsiella pneumoniae* and *K. oxytoca* being the most common ESBL-E. Additionally, 14 (12%) were CRE with *K. pneumoniae, E. coli* and *Enterobacter cloacae* complex being the most common CRE. Of CRE, 7 (50%) were carbapenemase producers with 4 (57%) KPC, 2 (29%) NDM and 1 (14%) KPC and NDM.

**Figure:**
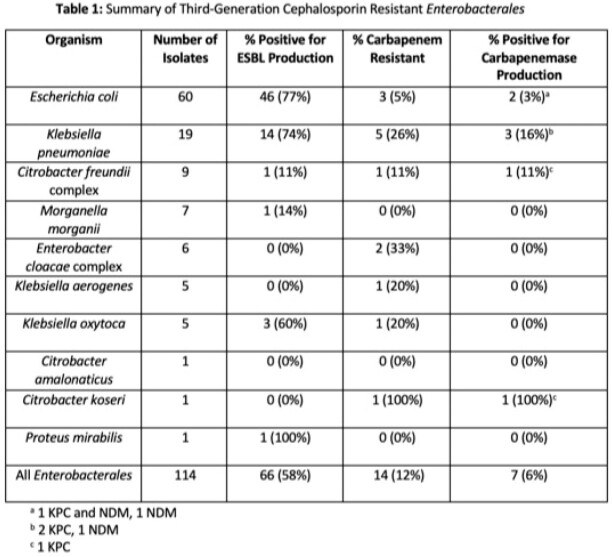

**Conclusion:**

Colonization with 3GC-RE and CRE occurs in 10% and 1% of rectal swabs collected from high-risk patients, respectively. 58% of 3GC-RE were ESBL producers and ranged between 0-100% among 3GC-RE; including up to 33% from inducible AmpC producing *Enterobacterales*.

**Figure:**
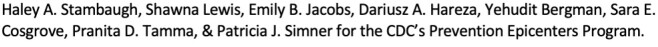

**Figure:**
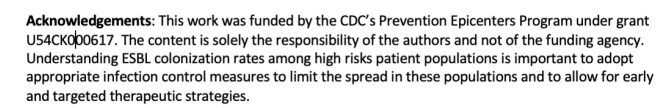

**Disclosures:**

**Sara E. Cosgrove, MD, MS**, Debiopharm: Advisor/Consultant|Duke Clinical Research Institute: Advisor/Consultant **Patricia J. Simner, PhD**, Affinity Biosensors: Grant/Research Support|BD Diagnostics: Advisor/Consultant|BD Diagnostics: Grant/Research Support|Entasis: Advisor/Consultant|GeneCapture: Stocks/Bonds|Merck: Advisor/Consultant|OpGen Inc: Board Member|OpGen Inc: Grant/Research Support|OpGen Inc: Honoraria|Qiagen Sciences Inc: Advisor/Consultant|Qiagen Sciences Inc: Grant/Research Support|Shionogi Inc: Advisor/Consultant|T2 Biosystems: Grant/Research Support

